# Development of a Novel Mobile App on Emergency Management Among Patients With Acute Ischemic Stroke at County-Level Hospitals in China

**DOI:** 10.2196/76311

**Published:** 2026-03-27

**Authors:** Qikai Wang, Conghua Fan, Yan Gu, Wen Zuo, Hu Lv, Danyang Yang, Libing Yun, Zhi Yan

**Affiliations:** 1 West China School of Basic Medical Sciences and Forensic Medicine, Sichuan University, No. 17, Section 3, Renmin South Road, Chengdu, 610044, China, 86 13880613969; 2 Xichang People’s Hospital, Xichang, China; 3 School of Medical Technology, Sichuan College of Traditional Chinese Medicine, No.1, Education Middle Road, Mianyang, 621000, China, 86 13681482661; 4 Capital Medical University, Beijing, China

**Keywords:** acute ischemic stroke, mobile app, emergency management, door to needle time, county-level hospitals

## Abstract

This retrospective study of 428 patients with acute ischemic stroke at a county-level hospital in China found that implementing the Xheart novel mobile app significantly reduced the median door-to-needle time from 52 to 38 minutes (*P*<.001) and was associated with lower National Institutes of Health Stroke Scale scores 24 hours after thrombolysis (*P*=.02), indicating the potential of mobile health technologies to improve the emergency management of patients with acute ischemic stroke in resource-constrained settings.

## Introduction

Stroke has become a main cause of death and disability-adjusted life-year loss in China [1], and ischemic stroke accounts for more than 80% of all incident strokes [2]. Shorter door-to-needle times (DNT) are associated with better acute ischemic stroke (AIS) outcomes, but guideline-recommended DNT (<60 min) is rarely achieved in county-level hospitals due to limited resources and fragmented communication [3,4]. Mobile technologies have the potential to shorten DNT and improve the prognosis of patients with AIS [5,6], but reports from resource-constrained settings are limited. We aimed to explore the effects of a new mobile app on the emergency management of patients with AIS in county-level areas in China.

## Methods

### Participants

This retrospective study was conducted at Xichang People’s Hospital between March 1, 2019, and December 31, 2024. The participants included 428 patients who consecutively presented to the emergency department with AIS within 6 hours of its onset. The exclusion criteria were as follows: (1) stroke happened in the hospital; (2) another stroke etiology such as cerebral hemorrhage or nonvascular causes; (3) patients without complete medical records.

### Mobile App

Xheart is a novel medical mobile app that has been freely available to 35 county-level hospitals in Sichuan since August 2020. The app comprises three core components: real-time information collection technology, real-time sharing of information technology, and process quality control management technology.

### Data Analysis

The data were analyzed using SPSS (version 26.0; IBM Corp). The Shapiro-Wilk test was used to check for normal distribution. Differences in continuous variables were analyzed using the Mann-Whitney *U* test, and differences in categorical variables were compared using the *χ*^2^ test. Univariable and multivariate linear regression analysis were conducted to assess associations between the new mobile app and the emergency management of patients with AIS. For all analyses, 2-sided *P* values <.05 were considered statistically significant.

### Ethical Considerations

This study was approved by the ethics committee of the Xichang People’s Hospital (ID: 2025‐50). The requirement for informed consent was waived and approved by the ethics committee. No compensation was provided to participants and all data were pseudonymized.

## Results

Characteristics and time metrics of participants are shown in Table 1. All recorded time intervals were significantly shorter in the post-app group (all *P*<.001). Particularly noteworthy were the median reductions in critical time intervals: DNT was reduced by 26.9% (38 vs 52 min, *P*<.001). Table 2 shows the associations between the mobile app and time metrics and outcomes. Multivariate linear regression showed that after adjustment for gender, age, National Institutes of Health Stroke Scale (NIHSS) score on admission, and onset to door time, the associations between the mobile app and shorter door to blood sample time (B=−4.02, 95% CI −5.03 to −3.00; *P*<.001), door to computed tomography results time (B=−3.55, 95% CI −5.10 to −2.01; *P*<.001), DNT (B=−15.34, 95% CI −19.7 to −10.98; *P*<.001), and lower NIHSS 24 hours after thrombolysis (B=−1.73, 95% CI −3.19 to −0.26; *P*=.02) remained statistically significant.

**Table 1.** Characteristics, time metrics, and clinical outcomes of participants.

Variable	Pre-app group (n=52)	Post-app group (n=376)	Z-score/chi-square^a^	*P* value
Male, n (%)	27 (52.0)	252 (67.0)	4.59	.03
Age (y), mean (range)	68 (61-77)	68 (58-76)	−0.50	.62
Onset to door time, mean (range)	99 (52-145)	166 (127-229)	−6.90	<.001
Door to neurologist time, mean (range)	5 (3-8)	3 (1-5)	−3.62	<.001
Door to blood sample time, mean (range)	10 (9-13)	6 (5-8)	−9.24	<.001
Door to CT^b^ time, mean (range)	11 (10-14)	9 (8-12)	−4.13	<.001
Door to CT results time, mean (range)	19 (17-21)	14 (12-17)	−6.75	<.001
DNT^c^, mean (range)	52 (42-58)	38 (31-46)	−5.88	<.001
NIHSS^d^ score on admission, mean (range)	9 (4-15)	4 (2-10)	−4.51	<.001
NIHSS score after thrombolysis, mean (range)	7 (4-15)	3 (1-9)	−4.90	<.001
NIHSS score 24 h after thrombolysis, mean (range)	5 (3-13)	2 (1-7)	−4.05	<.001
NIHSS score improvement after thrombolysis, mean (range)	0 (0-1)	0 (0-1)	−0.17	.87
NIHSS score improvement 24 h after thrombolysis, mean (range)	1 (0-4)	0 (0-2)	−1.77	.08

a All values are *z*-scores except the “Male” row (chi-square test, *df*=1).

b CT: computed tomography.

c DNT: door-to-needle time.

d NIHSS: National Institutes of Health Stroke Scale.

**Table 2.** Associations between the mobile app and time metrics and outcomes. Multivariate model, adjusted for male gender, age, NIHSS^a^ score on admission, and onset to door time.

Variable	Univariate model		Multivariate model	
	B (95% CI)	*P* value	B (95% CI)	*P* value
Door to blood sample time	−4.33 (−5.27 to −3.38)	<.001	−4.02 (−5.03 to −3.00)	<.001
Door to neurologist time	−1.16 (−2.34 to 0.02)	.05	−0.96 (−2.24 to 0.31)	.14
Door to CT^b^ time	−1.46 (−2.65 to −0.28)	.02	−1.21 (−2.48 to 0.06)	.06
Door to CT results time	−3.68 (−5.13 to −2.24)	<.001	−3.55 (−5.10 to −2.01)	<.001
DNT^c^	−13.99 (−18.04 to −9.94)	<.001	−15.34 (−19.7 to −10.98)	<.001
NIHSS score after thrombolysis	−4.14 (−5.99 to −2.30)	<.001	−0.51 (−1.33 to 0.31)	.22
NIHSS score 24 h after thrombolysis	−4.66 (−6.67 to −2.66)	<.001	−1.73 (−3.19 to −0.26)	.02

a NIHSS: National Institutes of Health Stroke Scale.

b CT: computed tomography.

c DNT: door-to-needle time.

## Discussion

### Principal Findings

Our study found that after the introduction of the Xheart mobile app, median DNT decreased significantly from 52 to 38 minutes. The mobile app could improve the emergency management of patients with AIS by RFID-based time tracking, real-time information sharing, and process monitoring. Previous studies have demonstrated the effectiveness of mobile technologies in the emergency management of patients with AIS [5-7]. Our study is unique in focusing on resource-limited county-level settings in China. Integrating mobile health technologies with predictive models [8-10] could further optimize real-time risk assessment and patient stratification, offering a practical strategy to improve emergency management for patients with AIS in underserved areas.

### Limitations

Limitations include retrospective single-center design, small pre-app sample size, and unmeasured confounding factors such as concurrent improvements in doctors’ experiences. Besides, the lack of 90-day modified Rankin Scale data limits the assessment of clinical relevance.

### Conclusions

These findings contribute to the potential of mobile health technologies to address critical treatment delays in resource-constrained settings. This novel mobile app can serve as a paradigm for advancing intelligent medical products, thereby facilitating the expansion of analogous apps to enhance system integration efficiency.

## Abbreviations

AIS: acute ischemic stroke
DNT: door-to-needle time
NIHSS: National Institutes of Health Stroke Scale
