# Correction: Development of a Novel Mobile App on Emergency Management Among Patients With Acute Ischemic Stroke at County-Level Hospitals in China

**DOI:** 10.2196/101442

**Published:** 2026-08-10

**Authors:** Qikai Wang, Conghua Fan, Yan Gu, Wen Zuo, Hu Lv, Danyang Yang, Libing Yun, Zhi Yan

**Affiliations:** 1 West China School of Basic Medical Sciences and Forensic Medicine, Sichuan University, No. 17, Section 3, Renmin South Road, Chengdu, 610044, China; 2 Xichang People’s Hospital, Xichang, China; 3 School of Medical Technology, Sichuan College of Traditional Chinese Medicine, No.1, Education Middle Road, Mianyang, 621000, China, 86 13681482661; 4 Capital Medical University, Beijing, China

In “Development of a Novel Mobile App on Emergency Management Among Patients With Acute Ischemic Stroke at County-Level Hospitals in China” [1], the authors noted one correction.

In the Funding section, the following sentence was revised:

*This study was supported by Xichang Science and Technology Project (JSYJ-2022‐14), Central Guided Local Science and Technology Development Fund Project (MXZB2025000822), and Research project of Sichuan Provincial Health Development Research Center (SCF24-C-100).*

This section now reads:

*This study was supported by Xichang Science and Technology Project (JSYJ-2022‐14), Central Guided Local Science and Technology Development Fund Project (2025ZYDF011), and Research project of Sichuan Provincial Health Development Research Center (SCF24-C-100).*

The correction will appear in the online version of the paper on the JMIR Publications website, together with the publication of this correction notice. Because this was made after submission to PubMed, PubMed Central, and other full-text repositories, the corrected article has also been resubmitted to those repositories.
